# Supplementary material for: Associations of Maternal Serum Iodine Concentration with Obstetric Complications and Birth Outcomes—Longitudinal Analysis Based on the Huizhou Mother–Infant Cohort, South China
Source: Nutrients. 2023 Jun 25;15(13):2868. doi: 10.3390/nu15132868 (PMC10346218; doi:10.3390/nu15132868)
Supplement: Supplementary file 1 [file nutrients-15-02868-s001.zip › nutrients-2389856-supplementary.pdf]

Supplemental Tables

Supplemental Table S1. Comparisons of baseline characteristics of pregnant women who were included for analysis in early pregnancy with those who were followed up for birth outcomes

	Women recruited during early pregnancy (n=1101)	Women followed-up till delivery (n=809)	*P*
Age（y）	28.1±3.3	28.2±3.2	0.246
Height（cm）	158.2±5.5	158.4±5.5	0.567
Pre-pregnant BMI（kg/m^2）	20.8±3.1	20.8±3.1	0.881
Education[a]			0.828
Primary school and below	4（0.4）	2（0.2）	
Junior middle school	196（17.8）	133（16.4）	
Senior high school	271（24.6）	193（23.9）	
College or vocational high school	354（32.2）	265（32.8）	
University and above	272（24.7）	216（26.7）	
Nulliparity，n（%）	599（54.4）	431（53.3）	0.610
Smoking，n（%）	28（2.6）	20（2.5）	0.918
Passive smoking, n (%)	521（47.4）	388（48.0）	0.796
Alcohol drinking，n（%）	34（3.1）	23（2.8）	0.753

Data were presented as mean±SD for continuous variables and compared by student t-test or n(%) for categorical variables and compared by chi-square test. BMI, body mass index.

Supplemental Table S2 Basic characteristics between women with delivery of small for gestational age (SGA) and those with appropriate for gestational age (AGA), Huizhou Mother-infant Cohort.

	AGA (n=689)	SGA (n=100)	*P*
Maternal age（y）	28.2±3.2	27.5±3.1	0.052
Height（cm）	158.5±5.4	157.0±6.1	<0.001
Pre-pregnant BMI(kg/m^2)	20.3±3.1	19.5±2.9	0.002
Education，n（%）			0.211
Junior meddle school and below	108（15.7）	23（23.0）	
Senior/occupational middle school	169（24.5）	19（19.0）	
College/vocational high school	227（32.9）	29（29.0）	
University or above	185（26.9）	29（29.0）	
Smoking，n（%）[a]	17（2.5）	3（3.0）	0.651
Alcohol drinking，n（%）[a]	23（3.3）	0（0.0）	0.102
Nulliparity，n（%）	353（51.2）	72（72.0）	<0.001
FT3, pmol/L	4.97（4.53, 5.43）	4.93（4.34, 5.42）	0.412
FT4, pmol/L	17.21（15.84, 18.70）	17.67（16.31, 19.16）	0.020
TSH, mIU/L	0.97（0.53, 1.67）	0.93（0.51, 1.59）	0.590
SI, μg/L	87.56（77.41, 96.44）	86.93（77.73, 100.89）	0.561

Data were presented as mean±SD for continuous variables and compared by student t-test or n (%) for categorical variables and compared by chi-square test. Abbreviations: BMI, body mass index; SI, serum iodine.

a：using Fisher's exact probability method by Chi-square testing

Supplemental Table S3 Sensitivity analyses among mothers of full-term delivery (n=772) on the associations of serum iodine during early and mid-pregnancy and change% with obstetric complications by multivariable linear regression

	Unstandardized coefficients B (95% CI)	Standardized coefficients β	*P*
Maternal log_{10} SIC during the first trimester (T1)			
Apgar score at 1 min	0.400 (0.000,0.801)	0.070	0.050
Gestational weight gain (kg)	-5.329 (-9.884, -0.773)	-0.085	0.022
Maternal log_{10} SIC during the second trimester (T2)			
Apgar score at 1 min	0.496 (-0.104, 1.096)	0.088	0.105
Gestational weight gain (kg)	-12.946(-19.857, -6.034)	-0.203	<0.001
Maternal SIC change% from T1 to T2			
Gestational weight gain (kg)	-0.043 (-0.080, -0.007)	-0.131	0.019
Birth weight (kg)	-0.003 (-0.005, 0.000)	-0.085	0.054
Birth length (cm)	-0.013 (-0.025, -0.001)	-0.092	0.038
Sensitivity analysis for SI change% by further adjustment of GDM status			
Gestational weight gain (kg)			
Birth weight (kg)	-0.002(-0.005, 0.001)	-0.068	0.123
Birth length (cm)	-0.012 (-0.024, 0.000)	-0.085	0.059

Multivariable linear regression models were applied with covariates being included in the models by enter methods. The adjusted covariates for gestational weight gain included maternal age (years), education (five categories from below primary school to above university), nulliparity (yes or no), pre-pregnancy BMI (kg/m^2), family history of diabetes (yes or no), and alcohol drinking (yes or no). The adjusted covariates for birth weight and length included maternal age (years), education (5 categories), prepregnant body mass index (kg/m2), delivery weeks (GWs), nulliparity (yes or no), neonatal gender (male or female), family history of diabetes (yes vs. no).

The adjusted covariates for Apgar score at 1 min included maternal age (years), education (5 categories), prepregnant body mass index (kg/m^2), nulliparity (yes or no), and birth weight (kg).
